# Responses at Individual Gamma Frequencies Are Related to the Processing Speed but Not the Inhibitory Control

**DOI:** 10.3390/jpm13010026

**Published:** 2022-12-23

**Authors:** Inga Griškova-Bulanova, Marko Živanović, Aleksandras Voicikas, Evaldas Pipinis, Vytautas Jurkuvėnas, Jovana Bjekić

**Affiliations:** 1 Institute of Biosciences, Life Sciences Centre, Vilnius University, Saulėtekio av. 7, LT-10257 Vilnius, Lithuania; 2 Institute of Psychology and Laboratory for Research of Individual Differences, Department of Psychology, Faculty of Philosophy, University of Belgrade, 11000 Belgrade, Serbia; 3 Department of General Psychology, Vilnius University, Universiteto 9/1, LT-01513 Vilnius, Lithuania; 4 Human Neuroscience Group, Institute for Medical Research, University of Belgrade, 11000 Belgrade, Serbia

**Keywords:** individual resonant frequency, gamma, cognitive performance, inhibition, auditory steady-state response (ASSR), envelope-following response (EFR)

## Abstract

The link between the state of networks underlying the generation of periodic responses at gamma ranges and cognitive outcomes is still poorly understood. In this study, we tested the idea that the individual differences in the ability to generate responses to auditory stimulation at gamma frequencies may underlie the individual differences in the inhibitory control. We focused on the processing speed and accuracy in the Bivalent Shape Task (a cognitive inhibition task assessing attentional interference) and explored the relationship with responses at 40 Hz and at individual gamma frequencies (IGFs, assessed utilizing auditory envelope-following responses in 30–60 Hz range). In a sample of 70 subjects, we show that individual measures (phase-locking index and event-related spectral perturbation) of the ability to generate gamma-range activity are not related to the individual differences in inhibitory control but rather reflect basic information processing speed in healthy young subjects. With the individualized approach (at IGFs), the observed associations were found to be somewhat stronger. These findings have important implications for the interpretation of gamma activity in neuropsychiatric disorders.

## 1. Introduction

Electroencephalography (EEG) is an affordable and easy-to-implement technique for research and clinical settings. With the increasing availability and the use of EEG-based assessment in various neuropsychiatric disorders and psychological conditions, the search for individual sensitive biomarkers for correct diagnosis or treatment outcome monitoring of neuropsychiatric disorders becomes of great importance. A lot of attention has been paid to the EEG activity in the gamma range due to its involvement in cognitive processes [1] and impairment in neuropsychiatric conditions [2]. Along with poor performance on cognitive tests, individuals with neuropsychiatric disorders typically display reduced gamma oscillations [3,4].

The gamma-range auditory steady-state response (ASSR) is frequently employed in neuropsychiatric disorders to assess deficits in gamma generation potential, and results are often discussed in relation to changed cognitive functioning [3,5,6]. As gamma-range ASSRs reflect the imbalance between inhibition and excitation in the brain circuits [3,7,8], the alterations of ASSRs are observed already in ultra-high-risk subjects [9] and show the promise to track the response to treatment [10,11], thus being a potentially sensitive marker of conditions where subtle changes in the state could occur. However, the link between the state of networks underlying the generation of periodic responses at gamma ranges and cognitive outcomes is still poorly understood.

A recent systematic review showed that the majority of studies linking ASSR and cognitive performance were conducted in clinical populations, and the evidence of the relationship between cognition and ASSRs for normal functioning is lacking [12]. Thus, it remains unclear if ASSR is linked to deficiency or fundamental processes underlying cognition. In addition, previous studies employed a wide array of tasks often focusing on different cognitive functions, thus making it difficult to conclude if and which executive functions are related to gamma-range ASSRs [12]. Studies suggest that individual differences in the gamma-range ASSR to some extent may reflect the level of attentional control and the ability to temporarily store and manipulate the information in the working memory [12]. However, findings on the links between gamma-range ASSR and other core executive functions, such as inhibition or set-shifting [13], especially in healthy participants, are lacking.

Inhibition is one of the core domain-general executive functions that underlies complex cognitive processing. It reflects intentional overcoming and stopping of dominant, automatic responses [13], and it is in action when there are several simultaneously active conflicting processes. Cognitive inhibition underlies selective attention and attentional control, supports resistance to retroactive/proactive interference and distractions, and is usually in the service of aiding working memory [14]. Some findings show that gamma activity in response to periodic stimulation is related to cognitive performance in generic executive function tasks, such as those tapping planning and problem solving (Tower of London) [15]. Since these types of tasks are characterized by high levels of complexity and simultaneously capture different executive functions, it is difficult to disentangle which of the functions are reflected in gamma-range ASSR. Because the Tower of London, similarly to the Tower of Hanoi, mostly relies on the executive function of inhibition (due to goal–subgoal conflicts; see [13]), it can be expected that the association between gamma-range ASSR and performance underlies efficient conflict resolution. In experimental, and especially real-life contexts, cognitive inhibition is usually highly intertwined with working memory [14], so the use of relatively simple cognitive tasks evoking inhibition of prepotent tendencies free of memory demands such as typical interference tasks (e.g., Stroop test [16], Simon task [17], and Eriksen’s flanker task [18]) enables the relatively focal assessment of inhibition, and it may better reflect the relationship to oscillatory brain responses. In addition, these types of tasks allow the assessment of basic information processing such as cognitive processing speed.

It should be noted, however, that mostly single-frequency ASSRs (predominantly at 40 Hz) were employed in previous studies exploring the relationship between ASSRs and cognitive functioning. This potentially limited the assessment of the individual resonant properties of the system and might have contributed to the inconsistent results. The cortical phase-locked activity patterns in the gamma range strongly vary across individuals, including the preferred frequency of the entrainment [19,20]. The individual gamma frequency (IGF), characterized as the frequency of the maximal response to periodic stimulation, reflects the individual resonance in the gamma range. Thus, responses at IGFs may be more readily reflecting the state of neural networks that are important for information processing, and the neuronal information transfer efficiency in the gamma range may relate to the variation in behavioral performance [21], including variability in cognitive inhibition.

In this study, we tested the idea that the individual differences in the ability to generate responses at the gamma frequencies may underlie the individual differences in inhibitory control. As an index of inhibition, we focused on the processing speed and accuracy in the Bivalent Shape Task [22] and explored the relationship with properties of the responses to auditory click-based chirp-like sounds (30–60 Hz, further referred to as envelope-following responses (EFRs)), both at 40 Hz (to match the broader research area) and at IGFs (to utilize individualized assessment).

## 2. Materials and Methods

The research consisted of two separate sessions—cognitive assessment in which subjects completed the cognitive inhibition task and the EEG recording session where the sounds were presented.

### 2.1. Participants

A group of 70 young subjects without a reported history of psychiatric and neurological disorders participated in the study (40 females, 2 left-handed; *M* age ± *SD* = 26.07 ± 4.28). The hearing thresholds of all subjects were within the normal range (<25 dB HL at octave frequencies). Participants abstained from alcohol 24 h prior to the testing and did not consume nicotine and caffeine-containing drinks at least one hour prior to the experiment. The study was approved by the Vilnius Regional Biomedical Research Ethics Committee (no. 2020/3-1213-701), and all participants gave their written informed consent.

### 2.2. Inhibition Task

**Figure 1:**
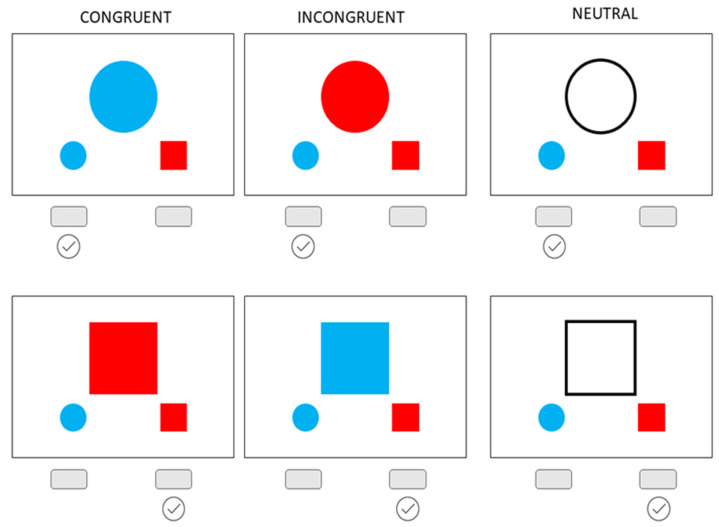
Bivalent Shape Task. Six stimuli were used in the task: the circle in blue color (congruent condition; correct response: left), red (incongruent condition, correct response: left), and no color (neutral condition; correct response: left), and the square in red color (congruent; correct response: right), blue (incongruent; correct response: right), and no color (neutral correct response: right).

The Bivalent Shape Task [22] is similar to different cognitive inhibition tasks assessing attentional interference (e.g., Stroop test [16], Simon interference task [17], and Eriksen’s flanker task [18]) but allows for a non-linguistic assessment of inhibitory control). In this task, participants are presented with a simple visual stimuli set up—one central shape (either circle or square) and two smaller shapes below (one circle and one square). In each trial, the participant is asked to determine if a shape presented at the center of the screen is a circle or a square and to simply press the left button if it is a circle and the right button if it is a square, as indicated by a visual response cue (i.e., smaller shapes) below each central shape (Figure 1). However, these response cues are colored in either red or blue, and the central shape is also presented either in red, blue, or only as a black outline. Therefore, there are three types of trials: (a) congruent condition—in which the color of the central shape matches the response cue color; (b) neutral condition—when the shape is presented only as an outline, i.e., a black line with no color fill; and (c) incongruent condition—in which the color of the central shape does not match the correct response cue but matches the incorrect one. The testing was performed using the Psychology Experiment Building Language [23].

As in a typical interference task, the participants are instructed to simply press the button to indicate the corresponding shape and disregard the color when deciding. There were 60 trials in total (20 per condition), and prior to the task, participants completed a short practice run consisting of 6 trials (2 per condition). The order in which the stimuli were presented was prerandomized and was the same for all participants.

We calculated the overall accuracy, i.e., the number of correct responses in the task (0–60), as well as the accuracy for each of the conditions (0–20). For each trial, the reaction time (RT) was recorded, and the average response time for all trials, as well as the average RT in each of the conditions, was calculated. The inhibition effect was calculated as the difference in the reaction times between the neutral and the incongruent condition for correctly responded trials. Thus, the RTs served as primary outcome measures, while the accuracy scores were used as secondary outcome from the Bivalent Shape Task.

### 2.3. EEG Acquisition

EEG was recorded with an ANT device (ANT Neuro, The Netherlands) and a 64-channel Wave Guard EEG cap (International 10–20 System) with Ag/AgCl electrodes. Mastoids were used as a reference; the ground electrode was attached close to Fz. Impedance was kept below 20 kΩ, and the sampling rate was set at 1024 Hz. Simultaneously, vertical and horizontal electro-oculograms (VEOG and HEOG) were recorded from above and below the left eye and from the right and left outer canthi.

### 2.4. Auditory Stimulation

The sounds were similar to previously used [24] click-based chirps. Stimulus train was created of single white-noise bursts spaced with changing inter-click periods to cover a range from 30 to 60 Hz in a descending order. The duration of the stimulus train was 720 ms, and 300 repetitions were presented with 700–1000 ms interstimulus intervals. The auditory stimuli were designed in the Matlab 2014 environment (The MathWorks, Inc., Natick, MA, USA) and presented binaurally through Shure SE215 earphones with sound pressure level adjusted to 60 dB with a DVM 401 dB meter (Velleman, TX, USA).

### 2.5. EEG Processing

The offline preprocessing of EEG data was performed in EEGLAB for MatLab© [25], as described previously [24]. The power-line noise was removed using multi-tapering and Thomas F-statistics (CleanLine plugin for EEGLAB). After the visual inspection and removal of channels with substantial noise (shift, movements), an independent component analysis (ICA) was performed with the ICA-implementation of EEGLAB (“runica” with default settings). Independent components related to eye movements (blinks and saccades) and EKG were removed.

Custom-written scripts based on EEGLAB and Fieldtrip functions [26] were used for further analysis steps. Epochs were created from −1000 ms to 2000 ms post-stimulus onset. Data were baseline-corrected to the mean of the prestimulus period, and epochs were further visually inspected for the remaining artifacts. Complex Morlet wavelet (7 cycles) from Matlab© Wavelet Toolbox with frequencies represented from 1 to 120 Hz, and 1 Hz intervals between each frequency, was used for wavelet transformation. Two measures of interest were extracted: the phase-locking index (PLI), reflecting the phase consistency over the trials, and the event-related spectral perturbation (ERSP corresponding to event-related changes in power relative to a prestimulus baseline) [27].

### 2.6. Envelope-Following Response

The baseline correction of the signal was performed by dividing the signal during the stimulation by the signal during the baseline averaged from −500 to −150 ms for each frequency. In accordance with previous studies utilizing chirps and classical gamma-range auditory steady-state responses, a clear fronto-central distribution of the response was observed [15,24]. For further evaluation, the PLIs and ERSPs at frequencies spanning 30–60 Hz were averaged for the fronto-central (Fz, Cz, FCz, C1, C2, F1, F2, FC1, and FC2) region where responses were most pronounced.

**Figure 2:**
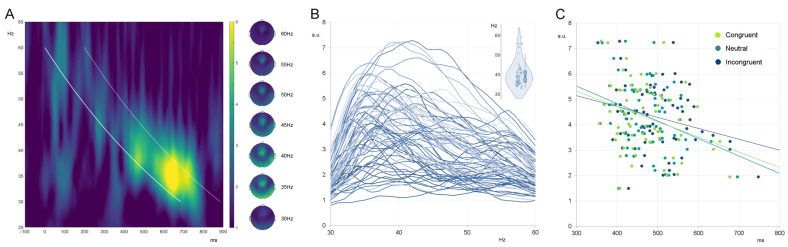
(**A**) The grand averaged time–frequency plot of the phase-locking index alongside the topographic activation plots at selected frequencies. (**B**) The individual phase-locking index curves and distribution of IGF. (**C**) Scatterplots of PLI values obtained at IGFs against reaction times in congruent, neutral, and incongruent conditions of the Bivalent Shape Task. a.u.—arbitrary units.

Following the proposed approach [24], responses to chirps were analyzed by extracting the curve representing time–frequency points of the stimulation. This curve was used to define the exact time points for each stimulation frequency (seen as a white bold line in the time–frequency plot in Figure 2). The average response was calculated to each stimulation frequency (from 30 to 60 Hz in 1 Hz steps) using a time window of +200 ms from the stimulation line (consistently with observed response windows in the time–frequency plots, seen as a white dashed line in Figure 2). The following individual indices were extracted and related to the individual outcomes on cognitive tasks: PLI/ERSP values at 40 Hz (40Hz_EFR) and maximal PLI/ERSP values (IGF_EFR).

### 2.7. Statistical Analysis

The statistical analysis was performed in JASP version 0.14.1 [28,29]. First, for the Bivalent Shape Task, we excluded all RTs for incorrect responses and responses shorter than 200 ms. Next, RTs were trimmed prior to analysis and the extreme RTs within each condition, i.e., the RTs falling outside the range of ± 3SD, were replaced with the lower and upper bound of the interval. The trimming was first performed between subjects and then within each subject.

To assess cognitive performance on the Bivalent Shape Task, we calculated descriptive statistics, i.e., mean (*M*), standard deviation (*SD*), and range (min–max) overall and for each condition separately. To assess the validity of the task, the differences in both accuracy and the response times were compared across conditions using the repeated measures ANOVA, with Holm-corrected post hoc tests. In the case of the sphericity violation (i.e., significant Mauchly’s W statistic), the Greenhouse–Geisser correction for unequal variances was applied. Moreover, in case the normality assumption was violated, the non-parametric Friedman test was conducted. In addition to the exact *p* values, we report measures of effect size—partial eta squared (η^2^_p_) for the omnibus test and Cohen’s *d* for post hoc comparisons.

The relationship between cognitive and EEG measures was assessed by Pearson correlation. The sensitivity analysis (the G*Power [30]) showed that with the sample size of 70 participants and the power of 0.95, correlations ≥ 0.235 could be detected at *p* ≤ 0.05 (two-tailed). To further assess the robustness of the effects, we employed the Bayesian inference approach. Namely, alongside correlations, we report the Bayes factor (BF_10_) that shows evidence for the alternative hypothesis (H_1_) relative to the null hypothesis (H_0_). The Bayes factors are interpreted as implemented in JASP, taking into account both evidence towards null and alternative hypotheses for correlations [31].

## 3. Results

### 3.1. Cognitive Performance

The descriptive statistics for the Bivalent Shape Task are presented in Table 1. The results show high overall accuracy (97.1% on average, range: 85.0–100.0%). Still, there were significant differences between conditions (F _(1.769,122.036)_ = 10.370, *p* < 0.001, and η^2^_p_ = 0.131), with lower accuracy in the incongruent condition (95.5%) in comparison with both congruent (98.5%, t = 4.416, *p* < 0.001, and d = 0.528) and neutral (97.5%, t = 2.665, *p* = 0.019, and d = 0.319), while the higher accuracy in congruent in comparison with the neutral condition was observed at the trend level (t = 1.716, *p* = 0.091, and d = 0.205). These results are corroborated by non-parametric tests of differences across conditions (χ^2^_(2)_ = 21.988 and *p* < 0.001).

The RTs were in the expected range (~500 ms), and with significant difference between the conditions (*F*_(1.833,126.476)_ = 25.837, *p* < 0.001, and η^2^_p_ = 0.272), as also observed in the non-parametric model (χ^2^_(2)_ = 24.886 and *p* < 0.001). Namely, the incongruent condition elicited longer RTs than both neutral (*t* = 4.370, *p* < 0.001, and *d* = 0.522) and congruent trials (*t* = 6.913, *p* < 0.001, and *d* = 0.826). The inhibition effect, i.e., the difference between RTs in neutral and incongruent trials, was, on average −18.85 ms (*SD* = 36.08). In addition, the shorter RTs were observed in the congruent in comparison with the neutral condition (*t* = 2.505, *p* = 0.015, and *d* = 0.299). As expected, the incongruency effect was of larger effect size than congruency, and there was a high correlation between RTs across conditions (congruent–neutral *r* = 0.916 and *p* < 0.001; congruent–incongruent *r* = 0.907 and *p* < 0.001; neutral–incongruent *r* = 0.894 and *p* < 0.001).

### 3.2. Envelope-Following Response

The descriptive statistics for the EFR are presented in Table 2.

The EEG responses resembled the expected fronto-central activation (Figure 2), being in line with previous observations [24]. The IGF ranged from 32 to 59 Hz, with a group mean of 40 Hz for PLI and 42 Hz for ERSP.

### 3.3. The Relationship between Cognitive and ERF Measures

The correlations between cognitive and ERF measures are shown in Table 3. No significant relationship was observed between accuracy (either overall or per condition) and any of the ERF parameters. Even more, the Bayesian analysis suggested moderate evidence in support of H_0_ (i.e., BF_10_ between 0.33 and 0.10) across the vast majority of accuracy correlations. On the other hand, the RTs were related to both PLI and ERSP measures, while no correlations were observed for the peak frequencies.

Interestingly, the correlations were observed for the overall RTs as well as for RTs in each condition, but the PLI and ERSP at IGF seemed to produce stronger and more reliable relations to RT than the same measures for 40 Hz. Specifically, the RTs in the congruent condition were significantly related to both PLIs and ERSPs at IGF, while the relationship was at the trend level for 40 Hz measures. For the neutral condition, the correlations were slightly higher at IGF than at 40 Hz, while in the incongruent condition, significant correlations were observed at IGF but not at 40 Hz. This resulted in overall more convincing correlations between overall RTs and PLIs and ERSPs at IGF than the same measures at 40 Hz. Altogether, the Bayesian analysis provided greater support for IGF-related correlations than the correlations with 40 Hz measures.

Finally, no correlations were observed between the inhibition measure and any of ERF measures. Namely, the individual differences in the inhibition effect correlated neither with PLI/ERSP measures at 40 Hz nor at IGF. The absence of the relationship received further support from the Bayesian analysis, which indicated moderate evidence towards H_0_.

## 4. Discussion

To the best of our knowledge, this is the first attempt to relate individual measures of the ability to generate gamma-range activity to individual performance on cognitive inhibition tasks. We recorded responses to auditory stimulation covering the frequency range within 30–60 Hz in a manner that was carried out in [24]. This allowed us to estimate IGFs as the stimulation frequencies producing the strongest and most synchronized responses [19] which, in accordance with previous reports [19,20], were around 40–42 Hz on the group level and in the 32–59 Hz range on the individual level. Responses showed topographical distribution with a clear fronto-central activation (Figure 2), thus being in accordance with classical single-frequency ASSRs [15]. To enable comparison with the existing 40 Hz ASSR literature, we assessed the phase-locking index and event-related spectral perturbation at 40 Hz (40Hz_EFR). To evaluate the prospect of an individualized approach, the same measures were extracted at IGF (IGFEFR), defined as the frequency of the maximal response.

In a previous study, we observed a negative correlation between EFR outcomes and move times on the Tower of London task, showing that subjects with better synchronization properties were faster in performing planning/problem-solving tasks [24]. Since the Tower of London is a complex task of executive functioning that, primarily but not exclusively, relies on the executive function of inhibition [13], it is difficult to conclude whether the observed relationship between EFR and RT reflects the efficacy of inhibitory control or some other aspect of executive functions and information processing. Therefore, in this study, we assessed the individual differences in cognitive inhibition using a simpler cognitive task—the Bivalent Shape Task [22]—which allows for the assessment of both RT and accuracy of individual conditions, as well as a more focal measurement of inhibitory control. To our knowledge, this is the first report on the use of this task in a sample of young healthy adults in the context of gamma activity.

We assessed the association between the accuracy, speed, and index of inhibition with extracted EEG measures—individual frequencies, and ERSP/PLI measures at 40 Hz and at IGF. The results show that EEG parameters were unrelated to accuracy in any of the conditions. Evidence in favor of H_0_ was further supported by the Bayesian analysis. However, it should be noted that the accuracy measures for each condition and overall score were extremely high and exhibited extreme restriction of variance, although demonstrating the expected effect of differences between neutral, congruent, and incongruent conditions. This restriction of range most certainly diminished the possibility of accuracy measures in achieving correlations with any external measure, due to the mere lack of variability. Thus, the obtained findings should be taken with caution since, due to low task difficulty, no confident conclusions on the relationship between accuracy with gamma-range parameters can be made. In contrast to accuracy measures, RTs for each condition, as well as overall RT, were negatively correlated with the EEG parameters. Importantly, it was found that the PLI and ERSP at IGF produced stronger and more reliable relations to RTs than the same measures for 40 Hz. RTs in all three conditions were negatively related to both PLI and ERSP measures extracted at IGF; however, only RTs to neutral stimuli were significantly related to PLI and ERSP values at 40 Hz, while the relationship between measures at 40 Hz and RTs to congruent stimuli was observed only at the trend level. In contrast, no correlation between PLI/ERSP at 40 Hz and RTs to incongruent stimuli was found. The Bayesian analysis provided the strongest support to the alternative hypothesis (H_1_) relative to the null hypothesis (H_0_) for the relationships between EFR parameters and RTs to neutral stimuli across different EEG measures. On the other hand, no consistency in support of H_1_ across different EEG measures was found for RTs in the congruent condition; namely, for PLI and ERSP at IGF, the results leaned towards H_1,_ while for PLI and ERSP at 40 Hz, they leaned towards H_0_. A similar but much less convincing pattern was found for the relationship between EFR parameters and RTs in the incongruent condition.

Overall, these observations of negative associations suggest that subjects with better gamma generation properties (stronger and more synchronized response) in this study were faster to execute behavioral responses on the task. However, this was primarily the case for neutral condition, i.e., the baseline condition, suggesting that when the complexity of the stimuli increases, whether by facilitating cognitive processing, which is the case in the congruent condition, or by introducing interference in incongruent condition, the relationship between EFR parameters and RT diminishes. Looking at the differences between congruent and incongruent conditions in relation to the baseline (i.e., neutral condition), we can see that the facilitation effect (congruent condition) was smaller than the interference effect (incongruent condition). In support of the conclusion that the relationship with EFR decreases with the increased complexity of the process, somewhat higher correlations and greater support for H_1_ was found for the congruent than incongruent condition. This implies that both PLI and ERSP reflect basic speed of processing rather than interference resolution or the level of cognitive facilitation. In line with this, ASSR measures at both IGF and 40 Hz proved to be unrelated to the inhibition effect.

These findings are in accordance with previous results showing a negative correlation between EFR outcomes and move times on the complex, inhibition-salient Tower of London tasks [15]. Namely, in that study, similarly to the current observations, the association between gamma-range ASSR and outcome measures was stronger for individualized than non-individualized measures. However, these effects, similarly to current findings, do not provide evidence that EFR parameters represent physiological manifestations of inhibitory control, but rather represent a mere reflection of time necessary to process simple information and execute response.

Here, we did not observe associations between any behavioral measures and IGFs. Previously, IGFs were demonstrated to reflect the individual ability to detect small and sudden changes in sound stimuli [32,33], i.e., to determine temporal resolution. In our previous study, we also did not observe the relationship of IGF to the time necessary to execute complex cognitive tasks of planning and problem solving [24]. However, we expected that IGF as a reflection of the state of neural networks defining individual gamma properties would relate to the temporal dynamics of less complex information processing. Based on the study findings, this seems not to be the case. It is important to note that methodological factors, such as the power of this and most previous studies, may be a limiting factor to observe the correlations if they are smaller than 0.30. Alternatively, the IGF might not be a measure sensitive enough to capture individual differences in cognitive processing.

Our current results, together with previous observations, hint at the importance of individual differences in the ability to respond at gamma ranges. The general concept is further supported by the individualized reaction of 40 Hz ASSRs to pharmacological manipulation—the reduction in ASSRs during psilocybin intoxication in our recent study was evident only in subjects with initially stronger ASSRs [34]. Similarly, in patients with schizophrenia who had larger baseline ASSRs, the more robust cognitive gains in response to targeted cognitive training were observed [11]. It is possible that the individual gamma activity that reflects a certain level of inhibition/excitation may stand as an index of the overall “adaptive integrity“ of the lower-level perceptual networks in the brain, and it is important for behavioral and clinical manifestations [11]. The ability to suppress irrelevant information while executing a task, being critical for successful goal-directed human behavior, is a function of the prefrontal cortex [35,36]. Although the main sources of auditory-evoked gamma activity are in the auditory cortex [37,38], recent evidence suggests a significant involvement of the prefrontal cortex in the gamma-range ASSR generation [39,40], providing a potential link between low-level and cognitive processes—in this case, the speed at which those processes are carried out.

## 5. Conclusions

Individual measures of the ability to generate gamma-range activity are not related to the individual differences in inhibitory control but rather reflect basic information processing speed in healthy young subjects. However, when complexity of the stimuli increases, whether by facilitating cognitive processing (congruent condition) or by introducing interference (incongruent condition), the relationship between EFR parameters and RT diminishes, implying that both PLI and ERSP measures of gamma response reflect basic speed of processing rather than interference resolution or the level of cognitive facilitation.

## Figures and Tables

**Table 1:** Descriptive statistics for Bivalent Shape Task (accuracy and RT).

	Accuracy			RT (ms)		
	M	SD	Range	M	SD	Range
Congruent condition	19.69	0.63	17–20	468.20	68.33	357.00–677.80
Neutral condition	19.50	0.86	15–20	476.44	60.22	363.55–696.67
Incongruent condition	19.10	1.09	14–20	495.29	77.7	353.05–747.66
Overall	58.29	1.84	51–60	479.70	66.53	364.77–706.70

**Table 2:** Descriptive statistics for Envelope-Following Response.

		Min	Max	M	SD
PLI	40Hz_EFR	1.11	7.22	3.79	1.4
	IGF_EFR	1.49	7.29	4.31	1.3
	IGF	33	59	39.9	6.1
ERSP	40Hz_EFR	0.98	1.7	1.18	0.1
	IGF_EFR	1.01	1.77	1.23	0.2
	IGF	32	58	42.1	6.6

**Table 3:** The relationship between cognitive and ERF measures.

			Peak Hz		IGF		40 Hz	
			PLI	ERSP	PLI	ERSP	PLI	ERSP
Accuracy	Congruent	*r*	0.024	0.129	−0.109	−0.043	−0.048	−0.089
		BF_10_	0.152	0.259	0.222	0.159	0.161	0.194
	Neutral	*r*	0.081	0.094	−0.070	−0.016	0.015	0.028
		BF_10_	0.186	0.200	0.176	0.151	0.150	0.153
	Incongruent	*r*	−0.167	−0.119	−0.057	−0.170	−0.104	−0.105
		BF_10_	0.379	0.240	0.166	0.394	0.214	0.216
	Overall	*r*	−0.053	0.017	−0.104	−0.124	−0.071	−0.080
		BF_10_	0.164	0.151	0.214	0.249	0.177	0.184
RT	Congruent	*r*	−0.071	−0.008	−0.312 **	−0.294 *	−0.215 †	−0.229 †
		BF_10_	0.176	0.150	4.390	2.272	0.708	0.844
	Neutral	*r*	−0.069	0.026	−0.316 **	−0.325 **	−0.237 *	−0.285 *
		BF_10_	0.175	0.153	4.792	5.953	1.002	2.473
	Incongruent	*r*	−0.127	−0.055	−0.254 *	−0.259 *	−0.155	−0.197
		BF_10_	0.255	0.165	1.364	1.493	0.332	0.552
	Overall	*r*	−0.095	−0.016	−0.301 *	−0.299 *	−0.205 †	−0.240 *
		BF_10_	0.202	0.151	3.441	3.322	0.616	1.067
Differential RT	Inhibition	*r*	0.158	0.162	0.021	0.017	−0.062	−0.052
		BF_10_	0.344	0.359	0.151	0.151	0.170	0.163

Note. Statistical significance marked: † *p* < *0*.10, * *p* < *0*.05, ** *p* < *0*.01.

## Data Availability

The data presented in this study are available on request from the corresponding author. The data are not publicly available due to privacy restrictions.

## References

[1] Bosman C.A., Lansink C.S., Pennartz C.M.A. (2014). Functions of Gamma-Band Synchronization in Cognition: From Single Circuits to Functional Diversity across Cortical and Subcortical Systems. Eur. J. Neurosci..

[2] Herrmann C.S., Demiralp T. (2005). Human EEG Gamma Oscillations in Neuropsychiatric Disorders. Clin. Neurophysiol..

[3] Tada M., Kirihara K., Koshiyama D., Fujioka M., Usui K., Uka T., Komatsu M., Kunii N., Araki T., Kasai K. (2020). Gamma-Band Auditory Steady-State Response as a Neurophysiological Marker for Excitation and Inhibition Balance: A Review for Understanding Schizophrenia and Other Neuropsychiatric Disorders. Clin. EEG Neurosci..

[4] Sugiyama S., Ohi K., Kuramitsu A., Takai K., Muto Y., Taniguchi T., Kinukawa T., Takeuchi N., Motomura E., Nishihara M. (2021). The Auditory Steady-State Response: Electrophysiological Index for Sensory Processing Dysfunction in Psychiatric Disorders. Front. Psychiatry.

[5] Kim S., Jang S.-K., Kim D.-W., Shim M., Kim Y.-W., Im C.-H., Lee S.-H. (2019). Cortical Volume and 40-Hz Auditory-Steady-State Responses in Patients with Schizophrenia and Healthy Controls. NeuroImage Clin..

[6] Koshiyama D., Thomas M.L., Miyakoshi M., Joshi Y.B., Molina J.L., Tanaka-Koshiyama K., Sprock J., Braff D.L., Swerdlow N.R., Light G.A. (2020). Hierarchical Pathways from Sensory Processing to Cognitive, Clinical, and Functional Impairments in Schizophrenia. Schizophr. Bull..

[7] Neklyudova A.K., Portnova G.V., Rebreikina A.B., Voinova V.Y., Vorsanova S.G., Iourov I.Y., Sysoeva O.V. (2021). 40-Hz Auditory Steady-State Response (ASSR) as a Biomarker of Genetic Defects in the SHANK3 Gene: A Case Report of 15-Year-Old Girl with a Rare Partial SHANK3 Duplication. Int. J. Mol. Sci..

[8] Sivarao D.V., Chen P., Senapati A., Yang Y., Fernandes A., Benitex Y., Whiterock V., Li Y.-W., Ahlijanian M.K. (2016). 40 Hz Auditory Steady-State Response Is a Pharmacodynamic Biomarker for Cortical NMDA Receptors. Neuropsychopharmacology.

[9] Tada M., Nagai T., Kirihara K., Koike S., Suga M., Araki T., Kobayashi T., Kasai K. (2016). Differential Alterations of Auditory Gamma Oscillatory Responses between Pre-Onset High-Risk Individuals and First-Episode Schizophrenia. Cereb. Cortex.

[10] Koshiyama D., Kirihara K., Tada M., Nagai T., Fujioka M., Ichikawa E., Ohta K., Tani M., Tsuchiya M., Kanehara A. (2018). Auditory Gamma Oscillations Predict Global Symptomatic Outcome in the Early Stages of Psychosis: A Longitudinal Investigation. Clin. Neurophysiol..

[11] Molina J.L., Thomas M.L., Joshi Y.B., Hochberger W.C., Koshiyama D., Nungaray J.A., Cardoso L., Sprock J., Braff D.L., Swerdlow N.R. (2020). Gamma Oscillations Predict Pro-Cognitive and Clinical Response to Auditory-Based Cognitive Training in Schizophrenia. Transl. Psychiatry.

[12] Parciauskaite V., Bjekic J., Griskova-Bulanova I. (2021). Gamma-Range Auditory Steady-State Responses and Cognitive Performance: A Systematic Review. Brain Sci..

[13] Miyake A., Friedman N.P., Emerson M.J., Witzki A.H., Howerter A., Wager T.D. (2000). The Unity and Diversity of Executive Functions and Their Contributions to Complex “Frontal Lobe” Tasks: A Latent Variable Analysis. Cogn. Psychol..

[14] Diamond A. (2013). Executive Functions. Annu. Rev. Psychol..

[15] Parciauskaite V., Voicikas A., Jurkuvenas V., Tarailis P., Kraulaidis M., Pipinis E., Griskova-Bulanova I. (2019). 40-Hz Auditory Steady-State Responses and the Complex Information Processing: An Exploratory Study in Healthy Young Males. PLoS ONE.

[16] Stroop J.R. (1935). Studies of Interference in Serial Verbal Reactions. J. Exp. Psychol..

[17] Simon J.R. (1969). Reactions toward the Source of Stimulation. J. Exp. Psychol..

[18] Eriksen B.A., Eriksen C.W. (1974). Effects of Noise Letters upon the Identification of a Target Letter in a Nonsearch Task. Percept. Psychophys..

[19] Zaehle T., Lenz D., Ohl F.W., Herrmann C.S. (2010). Resonance Phenomena in the Human Auditory Cortex: Individual Resonance Frequencies of the Cerebral Cortex Determine Electrophysiological Responses. Exp. Brain Res..

[20] Gransier R., Hofmann M., van Wieringen A., Wouters J. (2021). Stimulus-Evoked Phase-Locked Activity along the Human Auditory Pathway Strongly Varies across Individuals. Sci. Rep..

[21] van Es M.W.J., Schoffelen J.-M. (2019). Stimulus-Induced Gamma Power Predicts the Amplitude of the Subsequent Visual Evoked Response. NeuroImage.

[22] Esposito A.G., Baker-Ward L., Mueller S. (2013). Interference Suppression vs. Response Inhibition: An Explanation for the Absence of a Bilingual Advantage in Preschoolers’ Stroop Task Performance. Cogn. Dev..

[23] Mueller S.T., Esposito A.G. (2014). Computerized Testing Software for Assessing Interference Suppression in Children and Adults: The Bivalent Shape Task (BST). J. Open Res. Softw..

[24] Parciauskaite V., Pipinis E., Voicikas A., Bjekic J., Potapovas M., Jurkuvenas V., Griskova-Bulanova I. (2021). Individual Resonant Frequencies at Low-Gamma Range and Cognitive Processing Speed. J. Pers. Med..

[25] Delorme A., Makeig S. (2004). EEGLAB: An Open Source Toolbox for Analysis of Single-Trial EEG Dynamics Including Independent Component Analysis. J. Neurosci. Methods.

[26] Oostenveld R., Fries P., Maris E., Schoffelen J.-M. (2011). FieldTrip: Open Source Software for Advanced Analysis of MEG, EEG, and Invasive Electrophysiological Data. Comput. Intell. Neurosci..

[27] Mørup M., Hansen L.K., Arnfred S.M. (2007). ERPWAVELAB a Toolbox for Multi-Channel Analysis of Time-Frequency Transformed Event Related Potentials. J. Neurosci. Methods.

[28] Love J., Selker R., Marsman M., Jamil T., Dropmann D., Verhagen J., Ly A., Gronau Q.F., Šmíra M., Epskamp S. (2019). JASP: Graphical Statistical Software for Common Statistical Designs. J. Stat. Softw..

[29] JASP—A Fresh Way to Do Statistics. https://jasp-stats.org/.

[30] Faul F., Erdfelder E., Lang A.-G., Buchner A. (2007). G*Power 3: A Flexible Statistical Power Analysis Program for the Social, Behavioral, and Biomedical Sciences. Behav. Res. Methods.

[31] Wetzels R., Wagenmakers E.-J. (2012). A Default Bayesian Hypothesis Test for Correlations and Partial Correlations. Psychon. Bull. Rev..

[32] Purcell D.W., John S.M., Schneider B.A., Picton T.W. (2004). Human Temporal Auditory Acuity as Assessed by Envelope Following Responses. J. Acoust. Soc. Am..

[33] Baltus A., Herrmann C.S. (2015). Auditory Temporal Resolution Is Linked to Resonance Frequency of the Auditory Cortex. Int. J. Psychophysiol..

[34] Viktorin V., Griškova-Bulanova I., Voicikas A., Dojčánová D., Zach P., Bravermanová A., Andrashko V., Tylš F., Korčák J., Viktorinová M. (2022). Psilocybin—Mediated Attenuation of Gamma Band Auditory Steady-State Responses (ASSR) Is Driven by the Intensity of Cognitive and Emotional Domains of Psychedelic Experience. J. Pers. Med..

[35] Barbas H., Zikopoulos B. (2007). The Prefrontal Cortex and Flexible Behavior. Neuroscientist.

[36] Wallis J.D., Funahashi S. (2007). Prefrontal Representations Underlying Goal-Directed Behavior. Representation and Brain.

[37] Herdman A.T., Lins O., Van Roon P., Stapells D.R., Scherg M., Picton T.W. (2002). Intracerebral Sources of Human Auditory Steady-State Responses. Brain Topogr..

[38] Roß B., Picton T.W., Pantev C. (2002). Temporal Integration in the Human Auditory Cortex as Represented by the Development of the Steady-State Magnetic Field. Hear. Res..

[39] Farahani E.D., Wouters J., van Wieringen A. (2021). Brain Mapping of Auditory Steady-State Responses: A Broad View of Cortical and Subcortical Sources. Hum. Brain Mapp..

[40] Manting C.L., Gulyas B., Ullén F., Lundqvist D. (2021). Auditory Steady-State Responses during and after a Stimulus: Cortical Sources, and the Influence of Attention and Musicality. NeuroImage.

